# Elevated auxin and reduced cytokinin contents in rootstocks improve their performance and grafting success

**DOI:** 10.1111/pbi.12738

**Published:** 2017-05-16

**Authors:** Wei Li, Chu Fang, Sanalkumar Krishnan, Junmei Chen, Hao Yu, Angus S. Murphy, Emily Merewitz, Lorenzo Katin‐Grazzini, Richard J. McAvoy, Ziniu Deng, Janice Zale, Yi Li

**Affiliations:** ^1^ Department of Plant Science and Landscape Architecture University of Connecticut Storrs CT USA; ^2^ Department of Crop Science Michigan State University East Lansing MI USA; ^3^ Department of Plant Science and Landscape Architecture University of Maryland College Park MD USA; ^4^ College of Horticulture, Hunan Agricultural University Changsha Hunan China; ^5^ Plant Pathology Department, Institute of Food and Agricultural Sciences Citrus Research and Education Center University of Florida Lake Alfred FL USA

**Keywords:** auxin, cytokinins, rootstock, root growth and development, lateral buds release, grafting success rate

## Abstract

Plant grafting is an important technique for horticultural and silvicultural production. However, many rootstock plants suffer from undesirable lateral bud outgrowth, low grafting success rates or poor rooting. Here, we used a root‐predominant gene promoter (*SbUGT*) to drive the expression of a tryptophan‐2‐monooxygenase gene (*iaaM*) from *Agrobacterium tumefaciens* to increase auxin levels in tobacco. The transgenic plants, when used as a rootstock, displayed inhibited lateral bud outgrowth, enhanced grafting success rate and improved root initiation. However, root elongation and biomass of *SbUGT::iaaM* transgenic plants were reduced compared to those of wild‐type plants. In contrast, when we used this same promoter to drive *CKX* (a cytokinin degradation gene) expression, the transgenic tobacco plants displayed enhanced root elongation and biomass. We then made crosses between the *SbUGT::CKX* and *SbUGT::iaaM* transgenic plants. We observed that overexpression of the *CKX* gene neutralized the negative effects of auxin overproduction on root elongation. Also, the simultaneous expression of both the *iaaM* and *CKX* genes in rootstock did not disrupt normal growth and developmental patterns in wild‐type scions. Our results demonstrate that expression of both the *iaaM* and *CKX* genes predominantly in roots of rootstock inhibits lateral bud release from rootstock, improves grafting success rates and enhances root initiation and biomass.

## Introduction

Grafting is a technique by which tissues of different plants are combined so as to continue their growth together (Goldschmidt, 2014; Warschefsky *et al*., 2016). Grafting is also an essential tool in horticulture and silviculture that is widely used in asexual propagation. Desirable scions (the upper parts) can be grafted on rootstocks (the lower parts) that are adapted to certain soil conditions such as wet or dry soils, or resistant to soilborne pests and diseases (Hartmann and Kester, 1975; Song *et al*., 2013). In tree fruit production, grafting of scions to rootstocks is used to produce dwarf trees, enhance disease resistance, increase fruit yield and quality, combine production of multiple varieties on a single tree and enhance fertilization (Artlip *et al*., 2016). More recently, grafting has been extended from fruit trees to vegetables for enhancing resistances to biotic and abiotic stresses, improving water and nutrient uptake or increasing yield (Melnyk and Meyerowitz, 2015; Nakamura *et al*., 2016; Warschefsky *et al*., 2016; Zhao and Song, 2014).

However, many woody plant species with excellent rootstock characteristics are difficult to root from stem cuttings. For instance, it is difficult to induce adventitious rooting on stem cuttings from the apple cultivar ‘M.9’, which is commonly used as a dwarfing rootstock (Pawlicki and Welander, 1995; Zhu *et al*., 2001). A dwarf pear cultivar, BP10030, is cold hardy and graft compatible with most pear varieties but it is also difficult to root from stem cuttings (Zhu *et al*., 2003).

Also, the undesirable outgrowth of lateral buds from rootstocks after grafting is a common phenomenon. If lateral shoots originating from the rootstock are not suppressed or removed, healing of the graft union can be adversely affected. The rootstock's lateral shoots also compete with scions for light and nutrients, inhibiting scion growth (Daley and Hassell, 2014). Chemical treatments or manual removal may be used to eliminate lateral shoots from rootstock but these procedures are time‐consuming and expensive (Daley and Hassell, 2014).

Traditional breeding efforts have made impressive progress towards improving rootstock performance in numerous plant species, but continued improvement remains limited to selection of existing traits within the gene pool of rootstock cultivars (Cousins, 2005). Hybridization breeding is also limited, as some elite traits may be lost in the process (van Nocker and Gardiner, 2014; Pinto, 2015). Progeny production, through sexual crossing and subsequent selection, is a lengthy and labour‐intensive process that can take a decade to reach fruition. For perennial fruit trees, such as walnut, breeding cycles can be 20–30 years (Xiong *et al*., 2015). In contrast to traditional breeding, transgenic plant technology can be used to introduce completely new traits into rootstock lines and at a much faster rate, sometimes within months (Gambino and Gribaudo, 2012).

Lateral branching in plants is regulated by interactions between the phytohormones indole‐3‐acetic acid (IAA, auxin), cytokinin and strigolactone (Ferguson and Beveridge, 2009). It has been reported that apically derived auxin inhibits lateral bud outgrowth and cytokinin directly or indirectly stimulates bud outgrowth (Müller and Leyser, 2011). Insertion of the *Agrobacterium* gene *iaaM* gene that encodes a tryptophan‐2‐monooxygenase into plants has been shown to convert tryptophan to indole‐3‐acetamide. Indole‐3‐acetamide is then slowly converted by endogenous hydrolases to the active phytohormone indole‐3‐acetic acid (Sitbon *et al*., 1992). Cytokinin dehydrogenase (*CKX*) degrades the phytohormone cytokinin. Here, we report the use of a root‐predominant gene promoter sequence (*SbUGT*) to drive the expression of *iaaM* gene and an *Arabidopsis* cytokinin oxidase/dehydrogenase gene (*AtCKX2*, abbreviated as *CKX*) using tobacco as a model plant. The transgenic plants, when used as rootstock, displayed inhibited lateral bud outgrowth, enhanced grafting success rate and improved root initiation and biomass. The combined use of the auxin‐overproducing and cytokinin‐inactivating genes in roots represents an excellent strategy for rootstock improvement.

## Results

### The *SbUGT::iaaM* expression inhibited the outgrowth of lateral buds following decapitation

**Figure 1:**
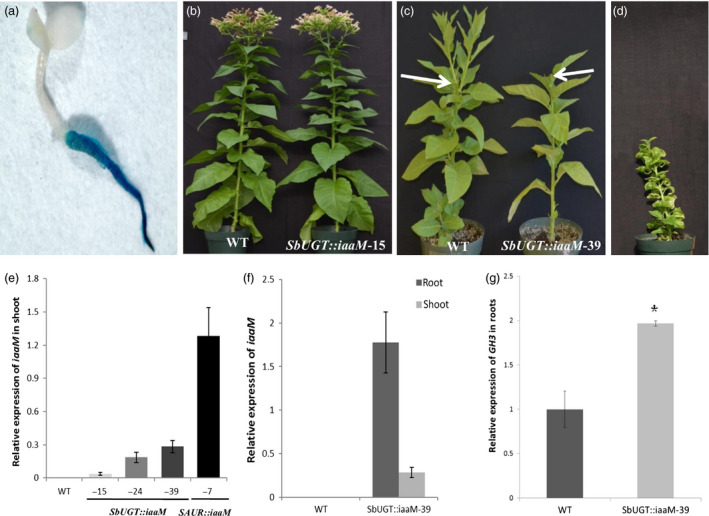
Root‐predominant expression of an auxin biosynthetic gene (*iaaM*) inhibited lateral bud release. (a) Histochemical staining of GUS activity in a 5‐day‐old *SbUGT::GUS* tobacco T_1_ seedling, showing that the *SbUGT* promoter was predominantly active in roots. (b) Four‐month‐old wild‐type and *SbUGT::iaaM*‐15 tobacco plants, showing that expression of the *SbUGT::iaaM* gene did not affect growth and developmental patterns of leaves and shoots. (c) Three weeks after decapitation, wild‐type plants released numerous lateral buds, while the *SbUGT::iaaM*‐39 plants had no lateral buds released from the decapitated shoots; the arrow heads indicate the decapitated shoots of wild‐type (left) and *SbUGT::iaaM*‐39 (right) tobacco. (d) A 4‐month‐old transgenic tobacco plant overexpressing the *iaaM* gene under the control of a shoot and leaf tissue active promoter (*SAUR*), displaying strong auxin‐overproducing phenotypes, reduced shoot elongation and leaf epinasty. (e) Expression levels of the *iaaM* gene in shoot tissues of 2‐month‐old *SbUGT::iaaM* and *SAUR::iaaM* tobacco plants. (f) Relatively high expression of the *iaaM* gene in roots but low expression in shoots was observed in the 2‐month‐old *SbUGT::iaaM*‐39 plant line. (g) Expression level of the auxin‐responsive *GH3* gene was enhanced in roots of *SbUGT::iaaM*‐39 line. Asterisks (*) represent significant differences between wild‐type and *SbUGT::iaaM*‐39 tobacco using two‐tailed Student's t‐test with the pooled variance (*P* < 0.05). Bars represent standard errors.

The *SbUGT::GUS* fusion gene was predominantly active in roots of transgenic tobacco plants (Figure 1a). The *SbUGT* promoter sequence was used to control the expression of the *iaaM*‐coding sequence. Of 58 *SbUGT::iaaM* tobacco lines produced, more than 75% of these plants showed no difference in growth and developmental patterns in the above‐ground organs when compared to wild‐type plants (Figure 1b). The remaining 25% showed a weak but visible auxin‐overproducing phenotype characterized by slight downward‐curled and epinastic leaves (Figure 1c, *SbUGT::iaaM*‐39 plant on the right). In contrast, the expression of the *iaaM* under the control of a *small auxin up RNAs* (*SAUR*) gene promoter, which is highly active in shoots and leaves (Li *et al*., 1992, 1994), resulted in stunted shoot growth and strong leaf epinasity (Figure 1d; also see Guilfoyle *et al*., 1992). Expression of *iaaM* in shoots appeared to inhibit lateral bud release in rootstocks following decapitation, as *iaaM* expression levels (Figure 1e) positively correlated with lateral bud release delays of 6 weeks in *SbUGT::iaaM*‐39, 4 weeks in *SbUGT::iaaM*‐24 and 1 week in *SbUGT::iaaM*‐15 lines.

In *SbUGT::iaaM*‐39 transgenic plants, the *iaaM* gene was highly expressed in roots, but also detectable in shoot tissues (Figure 1f). This result is slightly differently from histochemical staining of GUS activity in young seedlings (Figure 1a), which suggests the activity of the *SbUGT* promoter could be developmentally regulated. In *SbUGT::iaaM*‐39 roots, free IAA levels increased about threefold compared to wild type (Table 1), and expression of the endogenous auxin‐responsive gene *GRETCHEN HAGEN 3* (*GH3*) was increased (Li *et al*., 1999) (Figure 1g). Free IAA level in *SbUGT::iaaM*‐39 shoots also increased about twofold compared to that of wild‐type plants, with 414.1 ng/g dry weight for *SbUGT::iaaM*‐39 and 156.2 ng/g dry weight for wild‐type plants. Based on these observations, the *SbUGT::iaaM*‐39 line was selected for further experimentation.

**Table 1:** Endogenous auxin contents in roots of WT, *SbUGT::iaaM*‐39 (*iaaM*), *SbUGT::CKX*‐64 (*CKX*) and the *SbUGT::iaaM*‐39/*SbUGT::CKX*‐64 (*iaaM*+*CKX*) hybrid plants

Plants	Root IAA content (ng/g DW) (mean ± SE)
Wild type	351.9 ± 7.6c
*iaaM*	946.4 ± 20.3a
*CKX*	172.2 ± 15.8d
*iaaM*+*CKX*	830.4 ± 33.4b

Data represent the average of three biological replicates. Each replicate consists of the pooled root samples from 10 plants. Values followed by the different letters are significantly different at *P* < 0.05 (ANOVA; LSD). SE, standard errors.

### *SbUGT::iaaM* gene expression suppressed rootstock's lateral bud release and improved grafting success rates

**Figure 2:**
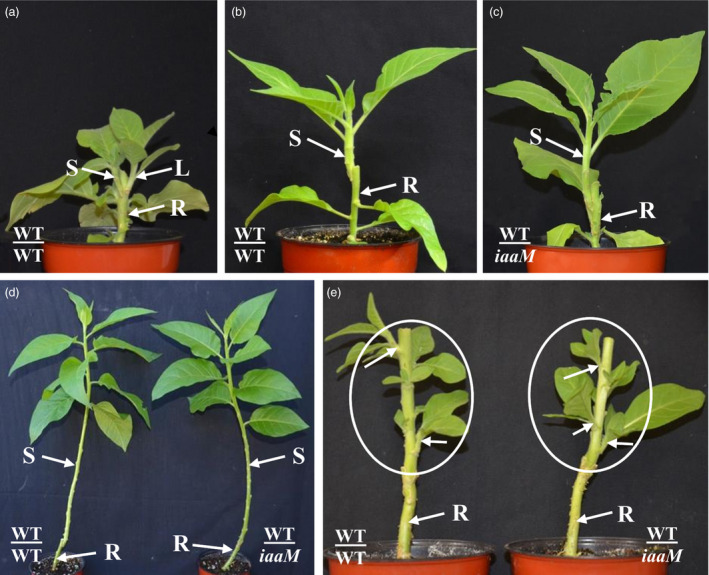
Using *SbUGT::iaaM*‐39 plants as rootstock led to inhibited lateral bud release from rootstock stumps and vigorous scion growth. (a) Two weeks after grafting, WT/WT grafts had released lateral buds and scion growth was inhibited. (b) If WT/WT grafts’ lateral buds were manually removed from the rootstock stumps, scions grew vigorously. (c) WT/*iaaM* grafts had no lateral buds released from the rootstock stumps, and scions grew vigorously. (d) Two months after grafting, WT/*iaaM* grafts had normal growth similar to WT/WT with lateral buds removed from rootstock stumps. (e) Two weeks following decapitation, scions of WT/*iaaM* had normal lateral buds release similar to the scions of WT/WT grafts, showing that increase in auxin in rootstock did not affect  normal growth and development of the scions. Circles show scions. Arrow heads indicate released lateral buds. S: scion. L: lateral bud. R: rootstock.

After wild‐type tobacco scions were grafted onto wild‐type plant rootstocks (abbreviated as WT/WT), lateral shoots began to develop from the rootstock within 2 weeks of grafting and the growth of scions was reduced (Figure 2a, 2 week after grafting, and Figure 4a, 3 weeks after grafting). When lateral buds were removed from wild‐type rootstock, vigorous scion growth was observed (Figure 2b). However, no lateral bud release from the rootstocks of WT/*SbUGT::iaaM‐39* (abbreviated as WT/*iaaM*) grafts was observed, and scion growth was also vigorous (Figure 2c). This was quite unlike scion growth of WT/WT grafts, which was vigorous only if the lateral buds of the rootstock were removed (Figure 2d). These results demonstrate that under these conditions, there is no need to remove lateral buds from rootstock, thus eliminating costs associated with that procedure.

When WT plants were used as rootstock, a 24% grafting success rate was observed if lateral buds were not removed from the rootstock (Table 2). After manual removal of buds from the WT rootstock, the grafting success rate increased to 68%. On the other hand, when the *iaaM* plants were used as rootstocks under the identical experimental conditions, we observed no lateral bud release and the grafting success rate reached 91%, demonstrating that expression of the *SbUGT::iaaM* in rootstock significantly enhanced grafting success rates.

**Table 2:** Grafting success rates of grafts with or without removing lateral buds from rootstock stumps

Grafting method	Grafting success rates (mean ± SE) (%)^a^			
	WT/WT (lateral buds intact on rootstock)	WT/WT (lateral buds removed from rootstock)	WT/*iaaM* (lateral buds intact on rootstock)	WT/*iaaM*+*CKX* (lateral buds intact on rootstock)
Decapitated plants as rootstock	24 ± 4a	68 ± 5b	91 ± 1c	89 ± 2c

a Grafts with more than 2‐cm increase in scion growth were considered as successful grafting. Data were collected 3 weeks after grafting. Each type of grafting has three replicates. For each replicate, 8–11 grafts were performed.

Values with the different letters are significantly different at *P* < 0.05 (ANOVA; LSD). SE, standard errors.

We also examined growth performance of WT/WT and WT/*iaaM* grafts under field conditions (Table 3). While lateral bud release was observed from the rootstock of the WT/WT grafts 10 days after planting, no lateral buds were released from the rootstock of the WT/*iaaM* grafts, demonstrating that the use of *iaaM* rootstocks can eliminate the need for lateral buds removal under field conditions. With lateral buds removed from the rootstock of the WT/WT grafts, the scions grew more vigorously, as indicated by height and dry biomass, than the WT/WT grafts for which lateral buds were intact. Scion growth in the WT/*iaaM* grafts that exhibited no lateral bud release was similar to that of the WT/WT grafts after manual lateral bud removal from the rootstock. Finally, lateral bud release from the scions of the WT/*iaaM* grafts was similar to that of the WT/WT grafts following apical shoot excision (Figure 2e), demonstrating that the *iaaM* rootstock had minimal effects on the branching behaviour of scions.

**Table 3:** Growth performance of scions of field‐grown grafts

Grafts (scion/rootstock)	Height on day 60 (cm)^a^ (mean ± SE)	Height on day 90 (cm)^b^ (mean ± SE)	Dry scion biomass (g)^c^ (mean ± SE)
WT/WT (lateral buds intact on rootstock)	66.4 ± 1.7a	99.8 ± 3.0a	69.9 ± 2.0a
WT/WT (lateral buds removed from rootstock)	76.3 ± 1.9b	128.4 ± 3.1b	83.8 ± 2.7b
WT/*iaaM* (lateral buds intact on rootstock)	81.2 ± 3.3b	127.8 ± 5.9b	81.5 ± 3.2b

a Height on day 60: plant height after 60 days in the field.

b Height on day 90: plant height after 90 days in the field.

c Dry scion biomass includes all stem and branch biomass above the graft union (excluding leaves); data were collected after 90 days in the field.

Data were collected from 10 individual plants and presented as averages. Values in the same column followed by the different letters are significantly different at p < 0.05 (ANOVA; LSD). SE, standard errors.

### The reduction in root growth observed in *SbUGT::iaaM* rootstock can be compensated by *SbUGT::CKX* expression

**Figure 3:**
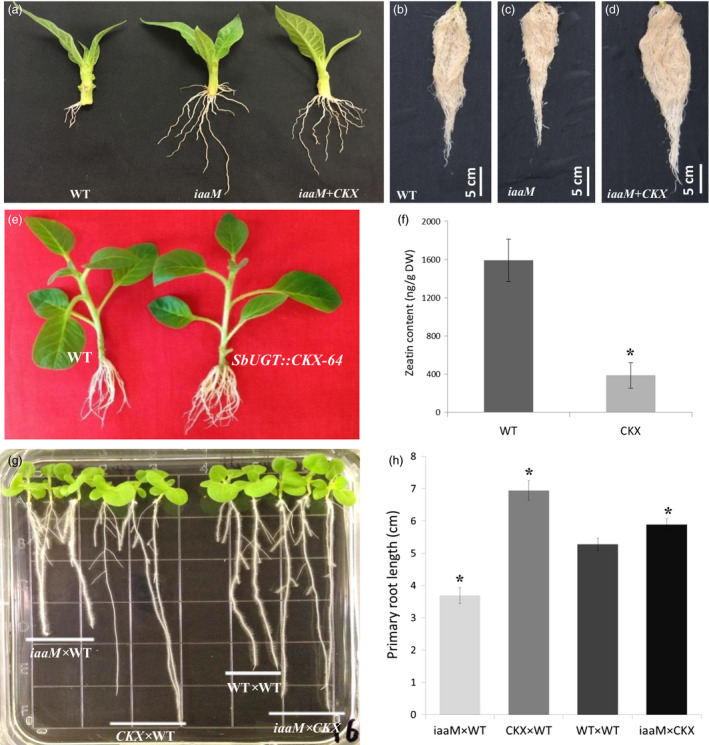
Negative effects of the *SbUGT::iaaM* gene expression on root growth can be compensated with expression of the *SbUGT::CKX* gene. (a) Stem cuttings with shoot tips of wild‐type, *SbUGT::iaaM*‐39 (*iaaM*) and *SbUGT::iaaM*‐39/*SbUGT::CKX*‐64 (*iaaM*+ *CKX*) hybrid plant after being rooted in fritted clay medium for 10 days, showing that the *iaaM* gene expression promotes root initiation. (b–d) Six‐week‐old (d) *iaaM*+ *CKX* plants had more root growth compared to the (b) wild‐type or (c) *iaaM* plants. (e) Stem cuttings of *SbUGT::CKX‐*64 (CKX) plants after being cultured on a MS medium for 3 weeks, showing more and longer roots than wild type. (f) Significant reduced zeatin contents observed in the roots of *CKX* plants when compared to wild‐type plants. (g) Eight‐day‐old progeny seedlings from the crossings of: *iaaM* or *CKX* to wild type, self‐crossed wild type and *iaaM* to *CKX*. The results showed that auxin‐mediated reduction in root growth was neutralized with expression of the *CKX* gene in roots. (h) Effects of the *iaaM* and *CKX* gene expression on primary root length. Asterisks (*) represent significant differences compared to wild type using two‐tailed Student's t‐test with the pooled variance (*P* < 0.05). Bars represent standard errors.

One concern about application of the *SbUGT::iaaM* expression as a practical technology was an observed reduction in root growth. Although root initiation in *iaaM* cuttings was more rapid compared to that of the wild‐type plants, root elongation and root biomass were reduced (Figure 3a–c, Table 4). To circumvent the negative effects of *iaaM* gene expression on root growth, we overexpressed an *Arabidopsis* cytokinin oxidase/dehydrogenase gene (*AtCKX2*, abbreviated as *CKX*) in roots. In general, *SbUGT::CKX* tobacco plants displayed improved root elongation and increased root biomass. Although this phenomenon was observed in multiple *CKX* overexpression lines, one line, *SbUGT::CKX‐64* (Figure 3e), was selected for further experiments. The roots of *SbUGT::CKX‐64* plants had significantly reduced endogenous cytokinin content compared to wild‐type plants (Figure 3f), demonstrating that expression of the *SbUGT::CKX* gene was effective at reducing cytokinin levels in roots.

**Table 4:** Growth characteristics of stem cuttings of *SbUGT::iaaM*‐39 (*iaaM*) and *SbUGT::iaaM*‐39/*SbUGT::CKX*‐64 (*iaaM*+*CKX*) plants under glasshouse conditions

Plants	Root number (mean ± SE)^a^	Root length (cm) (mean ± SE)^b^	Dry root biomass (mg) (mean ± SE)^b^	Shoot height (cm) (mean ± SE)^b^	Dry shoot biomass (mg) (mean ± SE)^b^
WT	4.4 ± 0.5	28.2 ± 0.7	543.6 ± 14.6	48.0 ± 0.9	3093.4 ± 146.1
*iaaM*	13.8 ± 1.1*	24.5 ± 0.9*	345.3 ± 23.9*	46.7 ± 1.5	2969.5 ± 73.25
*iaaM*+*CKX*	14.4 ± 1.2*	33.7 ± 1.0*	688.3 ± 57.3*	47.5 ± 1.2	3157.7 ± 55.3

a The average number of emerged roots per stem after being rooted in fritted clay medium for 10 days.

b Data were collected after being rooted in fritted clay medium for 6 weeks.

Data were collected from eight replicates and presented as averages. Asterisks represent significant differences compared to wild type using two‐tailed Student's *t*‐test with the pooled variance (*P* < 0.05). Bars represent standard errors.

Crosses of *SbUGT::iaaM*‐39 with *SbUGT::CKX*‐64 produced hybrids (*iaaM+CKX*) with both the *iaaM* and *CKX* transgenes present in progeny plants. Analysis of IAA content in root tissue of plants from both the *SbUGT::iaaM*‐39 and *iaaM+CKX* hybrid transgenic lines showed that overexpression of the *iaaM* gene led to significant increases in auxin concentration but overexpression of the *CKX* gene reduced auxin concentrations with or without overexpression of the *iaaM* gene (Table 1), similar as previously reported in *Arabidopsis* (Jones *et al*., 2010). These results demonstrate that expression of the *SbUGT::CKX* gene results in reduced IAA content in roots. The *SbUGT::CKX* overexpression‐mediated reduction in root auxin content may contribute to the improvement in root elongation and root biomass in *iaaM+CKX* hybrid plants.

Improvement of root elongation was observed in the seedlings derived from *iaaM*+*CKX* hybrid seed (Figure 3g, h). Rooting of shoot cuttings from *iaaM*+*CKX* hybrid plants after 10 days was also improved compared to wild‐type plants (Figure 3a). Six weeks after rooting, the *iaaM*+*CKX* hybrid plants produced longer roots than both *SbUGT::iaaM‐39* and wild‐type plants (Figure 3b–d). Dry root biomasses of the *iaaM*+*CKX* hybrid progeny plants were significantly greater compared to those of wild‐type plants. Shoot heights and dry shoot biomasses were similar between the *iaaM*+*CKX* and wild‐type plants (Table 4). These results demonstrate that reducing cytokinin levels in roots can neutralize the negative effects of root length and root biomass caused by the *iaaM* gene expression, and act synergistically with auxin to promote root initiation.

### Simultaneous expression of *SbUGT::iaaM* and *SbUGT::CKX* genes suppressed lateral bud release from rootstock and improved grafting success rates

**Figure 4:**
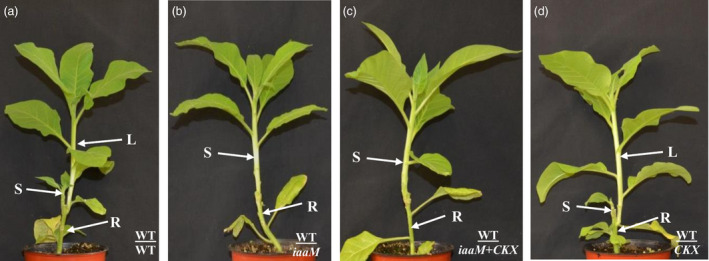
The *SbUGT::iaaM*‐39/*SbUGT::CKX*‐64 hybrid plant (*iaaM+CKX*) used as rootstock inhibited lateral bud release from their stumps and enhanced scion growth. (a–d) Three weeks after grafting, (a) WT/WT grafts had little scion growth because of outgrowth of a lateral shoot; (b) WT/*iaaM* and (c) WT/*iaaM+CKX* had no lateral buds released from their rootstock stumps and showed vigorous scion growth; and (d) WT/*CKX* had lateral shoot outgrowth with little scion growth**.** S: scion. L: lateral bud. R: rootstock.

If wild‐type scions were grafted on to wild‐type, *SbUGT::iaaM*‐39, *SbUGT::CKX*‐64 and *SbUGT::iaaM*+*SbUGT::CKX* rootstock, respectively, lateral buds were released from both the wild‐type rootstock (Figure 4a) and the *CKX* rootstock (Figure 4d), but not from the *iaaM* or the *iaaM*+*CKX* rootstock (Figure 4b and c). When lateral buds were released from the wild‐type or *CKX* rootstock, scion growth was inhibited (Figure 4a and d) but scion growth was vigorous when grafted onto the *iaaM* or the *iaaM*+*CKX* hybrid rootstock (Figure 4b and c). We also determined grafting success rates when *iaaM*+*CKX* hybrid plants were used as rootstock. Similar to that of the *iaaM*‐overexpressing rootstock, grafting success rate was dramatically improved when *iaaM*+*CKX* hybrid was used as rootstock relative to grafting success rate observed with wild‐type rootstock (Table 2). These results demonstrate that expression of both the *SbUGT::iaaM* and *SbUGT::CKX* genes in rootstock plants repressed lateral bud release from the rootstock and improved grafting success rate, similar to the effects of *SbUGT::iaaM* gene.

## Discussion

This study demonstrates that root‐predominant expression of an *iaaM* gene, whose product catalyses biosynthesis of an auxin precursor, results in a series of improved rootstock characteristics. However, root elongation and root biomass of *iaaM* rootstock were adversely affected compared to those of the wild‐type rootstock. We have further shown that overexpression of a cytokinin degradation gene (*CKX*) compensated the negative effect of the *iaaM* gene expression on root elongation and biomass of rootstocks. Our results have demonstrated that increases in auxin level and reductions in cytokinin concentration predominantly in roots can produce several beneficial characteristics including inhibited lateral bud release from the rootstock, improved grafting success rates and enhanced root initiation and root biomass. This technology may also be useful in other woody plants for improving the quality of rootstock because effects of auxin and cytokinin on plant growth and development are basically the same in higher plants. As such, the technology presented in this manuscript should be applicable for many economically important woody plants, such as apple and pear (Zhu *et al*., 2001, 2003). However, we also recognize that there are some differences in anatomical structure, developmental mechanisms and physiological characteristics among different plant species. Therefore, it is also possible that the effects of the root‐predominant expression of the *iaaM* and *CKX* genes may be somewhat different in woody plants than in tobacco.

Grafting success is largely dependent on the rapid formation of a graft union, where scion and rootstock fuse to form a chimeric plant. Yin *et al*. (2012) used an auxin‐responsive reporter gene (*DR5::GUS*) to show that endogenous auxin may accumulate in the graft union and therefore suggested a role for auxin in graft union formation. The increased auxin levels in the graft joint zone suggested by Yin *et al*. (2012) were most likely due to auxin accumulation in the basal end of the scion, as we have previously demonstrated (Li *et al*., 1999). However, more direct experimental evidence is needed to determine whether auxin plays an important role in grafting success rates. Using rootstocks that overexpress the *SbUGT::iaaM* gene, we have demonstrated that the WT/*iaaM* grafts had a much higher grafting success rate (91%) than the WT/WT grafts on which lateral buds were manually removed (68%). These results provide additional evidences that auxin plays a critical role in grafting success.

Lateral buds released from rootstock have been shown to negatively affect scion growth and thus grafting success. Lateral buds originating from the rootstock are usually removed manually (Daley and Hassell, 2014). Thimann and Skoog (1934) reported that prior to grafting, exogenously applied auxin to the rootstock could block the release of lateral buds. Chemical or manual removal of lateral buds from rootstock has also been used to eliminate lateral shoot development but these methods are time‐consuming and expensive (Choi *et al*., 2002; Memmott and Hassell, 2009). The transgenic approach presented here may provide an excellent tool to suppress lateral bud release from rootstock, improve grafting success rates and also reduce the costs associated with chemical or manual removal of lateral buds.

Fast initiation and establishment of adventitious roots from shoot cuttings are important traits for rootstock plants. However, many plant species or cultivars having a number of excellent rootstock characteristics are difficult to root. Dwarf apple rootstock varieties that have been commonly used for grafting are difficult to root from shoot cuttings (Pawlicki and Welander, 1995). Zhu *et al*. (2001) successfully used the *RolB* gene from *A. Rhizogenes* to enhance rooting of dwarf apple and pear varieties. The biochemical or molecular functions of the *RolB* gene in plants are still the subject of debate but some believe that the RolB protein may be involved in altering either hormone concentrations or signalling (Arshad *et al*., 2014). Similar to the results reported with *RolB* rootstock (Zhu *et al*., 2001), our auxin‐overproducing transgenic rootstock lines also displayed enhanced rooting ability. However, inhibition of lateral buds outgrowth from rootstock and improved grafting success rates were not reported with the use of *RolB* transgenic rootstock.

Reduction in cytokinin level has been shown to promote adventitious root initiation and elongation (Bellini *et al*., 2014). In *Arabidopsis*, transgene‐mediated reduction in endogenous cytokinin concentration, or mutations that alter the expression of cytokinin receptor genes, enhances adventitious root initiation (Riefler *et al*., 2006; Werner *et al*., 2003). Cytokinin may modify the expression of auxin transport genes such as *PIN* genes, reducing the formation of the auxin gradient required for the root initiation (Laplaze *et al*., 2007). Consistent with these previous reports, we have also observed that overexpression of the *SbUGT::CKX* gene alone or in combination with the *SbUGT::iaaM* gene enhanced root initiation and growth.

It has been reported that elevated auxin or cytokinin content in plants can effectively inhibit root elongation (Cary *et al*., 1995; Eliasson *et al*., 1989), while reductions in tissue cytokinin concentrations can promote root elongation (Werner *et al*., 2010). Werner *et al*. (2010) reported that a root‐specific reduction in the cytokinin concentration resulted in the development of longer primary roots. Rootstock plants with improved root length exhibited increased resistance to drought and nutrient stresses (Warschefsky *et al*., 2016). It will be interesting to determine whether the *SbUGT::CKX* rootstock plants also have enhanced tolerance to drought or nutrient stresses. Analyses for plant hormone concentrations in *SbUGT::iaaM* and *SbUGT::CKX* transgenic plants revealed that overexpression of the *CKX* gene resulted in reduced auxin levels. Jones *et al*. (2010) showed that cytokinins can enhance the expression of several *PIN* genes that are involved in cell‐to‐cell auxin transport, thus leading to altered auxin levels in the cell. However, whether the expressions of *PIN* genes in the *CKX*‐overexpressing transgenic plants are reduced still needs to be experimentally determined. It is, however, possible that lower auxin levels may have contributed to the improvement in root growth that we observed in the *CKX*‐overexpressing rootstock.

In this study, we have demonstrated that a differential expression of the *iaaM* and *CKX* genes can result in inhibition of lateral bud release from the rootstock, improved grafting success rates and enhanced root initiation and root biomass. Although transgenic technology provides a powerful tool for crop improvement, gene flow and food safety concerns over transgenic plants have impeded its utilization in the horticultural and forestry industries (Kausch *et al*., 2010; Li *et al*., 2016; Ye *et al*., 2016). The use of nontransgenic scions and transgenic *SbUGT::iaaM* and *SbUGT::CKX* rootstock may encounter less public opposition because fruits, seeds and pollen grains produced from scion shoots are nontransgenic.

## Experimental procedures

### Plasmid construction

The *SbUGT* promoter sequence, −102 to +86 relative to the transcription start site of a flavonoid glycosyltransferase gene from Scutellaria barbata (Chiou *et al*., 2010), was synthesized and inserted upstream of the *GusPlus‐*coding region in a pCAMBIA‐*GusPlus‐nptII* plasmid (Chen *et al*., 2006) to create the *SbUGT::GUS* construct. The *SbUGT* promoter sequence as well as the coding region of *iaaM* (a tryptophan‐2‐monooxygenase gene from *Agrobacterium tumefaciens*) (Sitbon *et al*., 1992) or *AtCKX2* (*Arabidopsis* cytokinin oxidase 2 gene) (Werner *et al*., 2003) were synthesized as one fragment and subcloned into a pCAMBIA*‐GusPlus‐nptII* plasmid to create the *SbUGT::iaaM* or *SbUGT::CKX* construct, respectively.

### Tobacco transformation and molecular confirmation of transgenic plants

Plasmid vector of *SbUGT::GUS, SbUGT::iaaM* or *SbUGT::CKX* construct was introduced into *Agrobacterium tumefaciens* strain EHA105, and the resulting bacteria were used to transform *Nicotiana tabacum* cv. Xanthi. Tobacco leaf disc transformation was performed as described previously (Zheng *et al*., 2007).

Genomic DNA was extracted from the leaves of putative transgenic plants using a modified CTAB protocol (Porebski *et al*., 1997). Extracted DNA was fractioned on a 0.8% (w/v) agarose gel in order to separate genomic DNA from any potential contamination from Ti‐plasmids. The purified genomic DNA was gel‐extracted and then used as template for PCR (Chen *et al*., 2006). The primer pair iaaM‐F (5′‐TTCTCCGAAGCACAACTA‐3′) and iaaM‐R (5′‐GCCCACCTAATGTCTCC‐3′) was used to amplify a 797‐bp fragment from the *iaaM* gene within the T‐DNA region of the Ti‐plasmid. The primer pair CKX‐F (5′‐CGTTATGGGTGGATGTG‐3′) and CKX‐R (5′‐TAAGCCAAGGATGAGGA‐3′) was used to amplify a 711‐bp fragment of the *CKX* gene within the T‐DNA region of the Ti‐plasmid. PCR reaction solution was 20 μL aliquot containing 1× PCR buffer (Takara, Japan), 1.5 mm MgCl_2_, 0.2 mm dNTPs, 0.2 μL e2TAK DNA polymerase (Takara, Japan), 0.25 μm of each primer and 500 ng DNA. The amplification started with an initial denaturation step at 98 °C for 5 min, followed by 35 cycles of 98 °C for 10 s, 60–65 °C for 5 s and 72 °C extension plus a final extension at 72 °C for 10 min.

### Histochemical GUS activity assays

T_0_ *SbUGT::GUS* tobacco plants were self‐pollinated to produce T_1_ progeny seeds. Five‐day‐old T_1_ seedlings were incubated in X‐gluc solution at 37 °C overnight for histochemical GUS activity staining. The histochemical assay staining solution contained 100 mm potassium phosphate buffer, pH 7.0, 10 mm Na_2_EDTA, 0.5 mm K_3_Fe(CN)_6_, 0.5 mm K_4_Fe(CN)_6_, 0.1% Triton X‐100 and 1 g/L X‐gluc (5‐bromo‐4‐chloro‐3‐indolyl‐β‐d‐glucuronic acid). Seedlings were treated with successive ethanol solutions, with increasing ethanol concentrations, to gradually remove chlorophylls and other pigments, after which they were then visually inspected and photographed.

### Quantitative real‐time PCR analysis

Shoot or root RNAs were extracted from 2‐month‐old *SAUR::iaaM*,* SbUGT::iaaM* or wild‐type tobacco plants using the RNeasy Plant Mini Kit including RNase‐Free DNase set (Qiagen, Valencia, CA) according to the manufacturer's protocol. The iScript™ cDNA Synthesis Kit (Bio‐Rad Laboratories, Richmond, CA) was used to synthesize cDNA, after which cDNA was used as a template for quantitative real‐time PCR analysis using SsoFast™ EvaGreen^®^ Supermix (Bio‐Rad Laboratories, Richmond, CA) on a CFX96™ Real‐Time PCR detection system (Bio‐Rad Laboratories, Richmond, CA). Primer sequences for all genes analysed are as follows:

iaaM forward: 5′‐TGGATTTCTCCGAAGCACA‐3′,
iaaM reverse: 5′‐CCCGGTAACGCATTTCAT‐3′,
GH3 forward: 5′‐GGATTATGCAATTTCAAGG‐3′,
GH3 reverse: 5′‐ACGATGGGCTAAAGTGTCT‐3′,
EF1α forward: 5′‐GCTGCTCAGAAGAAGAAATG‐3′,
EF1α reverse: 5′‐GAGCTGGTTCCAGACATACAC‐3′.

The tobacco *GH3* gene sequence was identified based on the deduced amino acid sequences from the *Arabidopsis* and soya bean *GH3* gene sequences. EF1α was used to amplify cDNA of the internal reference gene, elongation factor 1α (Schmidt and Delaney, 2010). Data were analysed using CFX Manager™ software version 2.0. The gene expression levels in each sample were normalized using the expression level of the elongation factor 1α gene in the same sample. Three biological replicates were performed with all treatments.

### Evaluation of *SbUGT::iaaM‐*39 rootstock in the glasshouse

The *SbUGT::iaaM‐*39 and wild‐type tobacco plants were vegetatively propagated and grew in glasshouse for one month before grafting. Scion and rootstock were jointed using the cleft graft technique (Lee and Oda, 2010). Parafilm was used to wrap the graft union for at least one week. A total of 20 WT/WT and 10 WT/iaaM grafts were used for each experiment. Of the 20 WT/WT grafts, 10 were left with the lateral buds intact on the rootstock and the other 10 had the lateral buds manually removed from the rootstock. The growth of the grafted plants was recorded two months after grafting. Two months after grafting, apical portions of the scions were removed, and lateral bud release from scion shoots was recorded after 2 weeks.

### Field evaluation of grafts

The *SbUGT::iaaM‐*39 and wild‐type tobacco plants were vegetatively propagated and grafted in the glasshouse as described above. A total of 20 WT/WT grafts and 10 WT/iaaM‐39 grafts were used for the experiment. Three weeks after grafting, all grafted plants were randomly planted in a field lot on the UConn depot campus in Storrs, Connecticut, USA, in July 2015. The 20 WT/WT grafts were divided into two groups: 10 with lateral buds on the rootstock intact and 10 with the rootstock lateral buds manually removed. Initial shoot heights of plants were recorded at time of transplanting in the field and then again at 60 and 90 days (October 2015). All scions above the graft union were harvested at day 90. After removing leaves, scion shoot tissues were oven‐dried at 70 °C for 10 days and then weighed. Shoot biomass was determined for each graft. Data were reported as means of all 10 replicates. Analysis of variance among field‐grown graft combinations was performed using IBM SPSS 19.0 (IBM Corp., Somers, NY). When sufficient differences (*P* < 0.05) were observed, Fisher's protected least significant difference test (*P* = 0.05) was performed to calculate differences between different treatments.

### Crosses between *SbUGT::iaaM‐*39 and *SbUGT::CKX*‐64 plants and hybrid progeny evaluation

Wild‐type*, SbUGT::iaaM‐*39 and *SbUGT::CKX*‐64 tobacco plants were vegetatively propagated. During flowering, wild‐type pollen were used to pollinate wild‐type, *SbUGT::iaaM‐*39 or *SbUGT::CKX*‐64 plants which had anthers removed before maturity to prevent undesired self‐pollination. *SbUGT::CKX*‐64 pollen was used to pollinate *SbUGT::iaaM‐*39 flowers in the same way. Paper bags were used to wrap pollinated flowers, in order to reduce undesired pollination. The progeny seeds were germinated and grown on MS medium. Genomic DNA was extracted from leaves of seedlings using a modified CTAB method (Porebski *et al*., 1997). The primer pairs, iaaM‐F and iaaM‐R or CKX‐F and CKX‐R (primer sequence information has been listed before), were used to confirm the presence of the *iaaM* or *CKX* genes in hybrid plants, respectively. Detailed information about primers has been described above. Eight days after germination, photographs and primary root length data were collected. Data were recorded on an average of 30 seedlings. Means between wild‐type and transgenic plants were compared using the two‐tailed Student's t‐test with the pooled variance (Steel et al., 1997).

### Root growth evaluation under glasshouse conditions

The *SbUGT::iaaM‐39*, wild‐type and one representative *iaaM+CKX* hybrid plant were vegetatively propagated and planted in pots with fritted clay medium in glasshouse. Ten days after planting, root number of each plant was recorded. Six weeks after rooting, shoot height of each plant was recorded. All plants were carefully dug out from medium. Root length was determined for each plant. Shoot and root tissues were oven‐dried at 70 °C for 10 days and then weighed. Data were reported as means of all eight replicates. Means between wild‐type and transgenic plants were compared using the two‐tailed Student's t‐test with the pooled variance (Steel et al., 1997).

### Evaluation of *iaaM+CKX* hybrid rootstock in glasshouse

The *SbUGT::iaaM‐39*, wild‐type and one representative *iaaM+CKX* hybrid plant were used as rootstock, and wild‐type scions were grafted as described above. One group of WT/WT grafts has lateral buds intact on the rootstock, and the other group has the rootstock lateral buds manually removed. Three weeks after grafting, grafting success rates were recorded. Grafts with more than a 2‐cm increase in scion's height growth were considered as successful grafts. For each rootstock/scion and lateral bud removal treatment, 8–11 grafts were performed as one replicate. Data were reported as means of three biological replicates. Analysis of variance on grafting success rates between different grafts was performed using IBM SPSS 19.0 (IBM Corp., Somers, NY). When sufficient differences (*P* < 0.05) were observed, Fisher's protected least significant difference test (*P* = 0.05) was performed to calculate differences between groups.

### Quantification of IAA and zeatin content

Hormone extractions were handled in the same manner as described (Krishnan and Merewitz, 2015; Krishnan *et al*., 2016). About 50 mg frozen‐dried root or shoot samples from two‐month‐old *SbUGT::iaaM‐39*,* SbUGT::CKX‐64,* one representative *iaaM+CKX* hybrid plant or wild‐type samples was ground to a fine powder in liquid nitrogen using a mortar and pestle. IAA or zeatin content analysis was carried out using an ultra‐high‐performance LC‐tandem mass spectrometer (UPLC/MS/MS) (Quattro Premier XE ACQUITY Tandem Quadrupole; Waters, Milford, MA). Samples from 10 plants were pooled for each replicate, and data were reported as a mean of three biological replicates. Analysis of variance was performed on IAA content data using IBM SPSS 19.0 (IBM Corp., Somers, NY). When sufficient differences (*P* < 0.05) were observed, Fisher's protected least significant difference test (*P* = 0.05) was performed to calculate differences between groups. Means of zeatin content between *SbUGT::CKX‐64* and wild‐type plants were compared using the two‐tailed Student's t‐test with the pooled variance.

## Author contributions

W.L. and C.F. performed most experiments. J.C. and H.Y. produced and initially characterized *SbUGT::CKX* tobacco plants. S.K. and E.M. were responsible for endogenous hormone analysis and manuscript editing. A.M., L.K.G., R.J.M., Z.D. and J.Z. provided suggestions on experiments and edited the manuscript. Y.L. designed the experiments and the organization of the manuscript. W.L. and Y.L. wrote and edited the manuscript.

## Conflict of interest

All authors declare that they have no conflict of interest.
